# Probiotics and Synbiotics Administered to Young Infants: Perceptions and Acceptability Amongst Carers and Healthcare Workers in Western Kenya

**DOI:** 10.3390/nu17030495

**Published:** 2025-01-29

**Authors:** Mary Iwaret Otiti, Florence Achieng Were, Sevim Zaim, Helen Nabwera, Simon Kariuki, Stephen Allen

**Affiliations:** 1 Department of Clinical Sciences, Liverpool School of Tropical Medicine, Pembroke Place, Liverpool, L3 5QA, UK; iwaret.otiti@lstmed.ac.uk (M.I.O.); helen.nabwera@aku.edu (H.N.); 2 Medical Research Institute (KEMRI) Centre for Global Health Research, Kisumu P.O. Box 1578 – 40100, Kenya; florenceachieng2005@gmail.com (F.A.W.); skariuki1578@gmail.com (S.K.); 3 Liverpool School of Tropical Medicine, Liverpool L3 5QA, UK; ssevimzaim@gmail.com

**Keywords:** malnutrition, probiotics, synbiotics, acceptability, perceptions, carers, Peer Mothers

## Abstract

Background/Objectives: A contributory factor to childhood undernutrition is poor gut health occurring within the first 6–12 weeks of life despite exclusive breastfeeding. Pro/synbiotic administration may protect gut health. A qualitative study was conducted amongst mothers/carers and healthcare workers (HCWs) to explore their perceptions and the acceptability of pro/synbiotics administration in early life. Methods: This study was nested within a randomised, open, clinical trial of pro/synbiotics with 32 doses administered under supervision to infants between ages 0 and 5 months in western Kenya. Semi-structured interviews were conducted with 14 mothers/carers, 12 Peer Mothers and 7 healthcare workers (HCWs) selected by purposive critical and key informant sampling. Interviews were transcribed and analysed using a thematic coding framework. Results: The satisfaction with the pro/synbiotic administration was very high amongst all three groups. Commonly perceived benefits included protection from diseases, healthy growth of the infant and improved appetite. The main barriers were working mothers and other commitments making it difficult to stick to scheduled administration visits, adverse judgement and opinions in the community, and a lack of engagement from fathers. Insights were gained into different means of administering pro/synbiotics to young infants. Triangulation of the findings of the mothers/carers with HCWs showed that most identified motivations and challenges were similar. Conclusions: Pro/synbiotic administration was well accepted by the mothers/carers and HCWs and generally perceived to have health benefits. The administration of pro/synbiotics by the mothers/carers themselves to their infants may be feasible and overcome logistical challenges. Greater efforts to sensitise and engage fathers and communities would likely be critical for a community-based program.

## 1. Introduction

Childhood undernutrition remains a global concern, with wasting estimated to affect 6.8% and stunting 22.3% of all children under 5 years of age in 2022. Ninety-seven percent of all wasted and 95% of all stunted children live in Asia or Africa [1]. Several nutrition-specific interventions, including promoting exclusive breastfeeding; nutritional supplements; immunomodulators; and improved water, sanitation and hygiene (WASH) have had a limited impact on preventing malnutrition [2,3,4]. This has renewed interest in the abnormal structure and function of the gut termed “environmental enteric dysfunction” (EED). EED is a sub-clinical condition characterised by damage to the small intestine and is thought to be due to colonisation of the gut by a range of enteropathogens as a result of poor sanitation and hygiene [5,6,7]. The major components of EED are inflammation of the intestinal mucosa, reduced surface area for nutrient digestion and absorption as a result of atrophy of the intestinal villi, and increased leakiness of the mucosal barrier [5,6]. EED likely impairs growth due to impaired nutrient digestion and absorption and increased systemic inflammation. EED can occur within the first 6–12 weeks, even amongst exclusively breastfed infants [8,9].

A potential novel approach to prevent or ameliorate EED is the administration of pro- or synbiotics in early life. Probiotics are live non-pathogenic microorganisms that when administered in adequate amounts, confer a health benefit on the host [10]. Probiotics may provide colonisation resistance against enteropathogens and also improve gut health through several mechanisms [11,12]. Prebiotics support the growth of beneficial microorganisms, conferring a health benefit to the host [13]. Synbiotics combine probiotics with prebiotics [14].

The administration of pro- and synbiotics to young infants in poorer communities may be feasible, safe and bring health benefits [15,16,17]. However, the perceptions regarding nutritional supplements and their acceptability amongst parents/carers and healthcare workers (HCWs), especially in exclusively breastfed infants, needs to be considered in the development of this intervention for community settings where undernutrition is common. The research question addressed in this study was “what are the perceptions, opinions and acceptability of parents/carers, Peer Mothers and HCWs regarding pro/synbiotic administration to infants in a low-resource setting in western Kenya?”

## 2. Materials and Methods

### 2.1. Study Setting and Population

This qualitative study was nested within the PRObiotics and SYNbiotics in infants in Kenya (PROSYNK) randomised, open-label trial in Homa Bay County, Nyanza Province, western Kenya (trial registration: PACTR202003893276712 [18]). The main aims of the PROSYNK study were to determine whether the administration of pro/synbiotics in newborns during the first 0–5 months of life would improve their gut health and growth. Stunting occurs in 13% of under-fives in this region [19]. Residents are predominantly of the Luo ethnic group, whose common livelihoods include agriculture, fishing and microenterprises.

Between October 2021 and January 2022, newborns delivered at the Homabay County Teaching and Referral Hospital with a birthweight ≥2000 g, who had taken at least one feed well and in whom there were no health concerns were recruited within the first 3 days of life. They were randomised into one of three groups (either a probiotic or one of two synbiotics) or a control arm (no intervention) according to a computer-generated random allocation sequence. Pro/synbiotics were presented as powder in capsules and were given once daily for the first 10 days and then weekly to age 6 months (a total of 32 visits/child). Each capsule was transported to the infant’s home in a cold box. After hand sanitising and using appropriate hygienic measures, the contents of the capsule could be sprinkled directly into the infant’s open mouth before feeding or mixed in a clean container with sterile water. Pro/synbiotic administration refers to the administration of either the probiotic or synbiotic powder contained within capsules to the infant either by the mother/carer or a member of the research team. All administration was supervised by a member of the research team. Mothers/carers also received support from trained Peer Mothers who were selected from the community after having participated in a previous study involving low birth weight babies [20]. Both the research staff and Peer Mothers advised mothers/carers on newborn and infant care practices during visits for pro/synbiotic administration. Community sensitisation was conducted prior to recruitment and enrollment into this study.

The compliance with the follow-up visits and pro/synbiotic administration was high, with 85% of home visits completed and 83% of doses administered successfully. Follow-up visits occurred mainly in the infants’ homes, with some pro/synbiotic administration occurring during routine hospital visits (e.g., for immunisation). Consistent with the Kenya national guidelines for research [21], face-to-face visits for the pro/synbiotic administration under supervision were maintained with the required precautions during the COVID-19 pandemic; however, follow-up was performed by mobile phone calls in the control arm during this period. Monitoring for serious adverse events was undertaken throughout this study, and none were attributed to the pro/synbiotic administration.

### 2.2. Sampling Procedure

This study was undertaken mainly in the rural community in Homa Bay County. Three participant subgroups were purposefully identified: (1) mothers/carers (mothers or carers of infants receiving or who had received a pro/synbiotic in PROSYNK and remained engaged in the trial); (2) HCWs (nurses and doctors from the county hospital and study clinicians); and (3) Peer Mothers. The recruitment of mothers/carers of infants enrolled in PROSYNK captured diverse perspectives and experiences at various stages of this study from recruitment to the completion of administration at age 6 months. HCWs were included to explore how pro/synbiotic administration could be implemented alongside recommended infant-feeding practices, including exclusive breastfeeding. Peer Mothers provided a perspective from the community. Mothers/carers, HCWs, and Peer Mothers who declined to give consent or were ill, as well as mothers/carers whose children were ill at the time of the interviews, were excluded.

### 2.3. Data Collection

Following an explanation of the study objectives and procedures and securing informed consent, semi-structured, in-depth interviews (IDIs) were undertaken during July 2021. The IDIs with mothers/carers and Peer Mothers took place in the community at a convenient time, which often coincided with home visits for the pro/synbiotic administration. The IDIs were performed in the language that the participants felt the most comfortable expressing themselves in (Kiswahili, Dholuo or English). The IDIs with HCWs at the hospital were conducted at a convenient time and mostly remotely via the WhatsApp mobile application, as this was during the COVID-19 pandemic period. A stable internet connection was established at the hospital and the IDIs were conducted in English. The interview questions were based on the topic guide generated through a discussion amongst the research team, reviewed by HCWs and pilot tested to confirm understanding (Table S1). The topics addressed during the IDIs included the acceptability of pro/synbiotics and administration processes, perceived benefits and barriers to the intervention, and knowledge of infant-feeding practices and infant care practices. The interviews were semi-structured, with the interviewer prompted by the topic guide.

### 2.4. Data Management and Analysis

The IDIs were recorded and stored on passcode-locked digital voice recorders and supplemented by field notes. The interviews were transcribed and translated into English by a research assistant who was a native speaker of both Kiswahili and Dholuo. All transcripts were reviewed by two study investigators (F.A., S.Z.) for clarity and translation quality, which allowed the investigators to inductively identify patterns prior to analytic coding.

The Health Belief Model [22,23] was chosen to achieve a better understanding of an individual’s approach to a health-related decision. The data analysis adopted the “thematic analyses method” approach for the systematic analysis of key and recurring themes. Although time constraints prevented triangulation using different methods of data collection, the patterns of convergence between the study groups were investigated during the analysis.

Audio recordings were transcribed using Expresscribe software (version 9.01) into an MS Word transcription template. Themes were generated first from the interview guide, and later, codes were developed from the responses. The thematic coding framework was developed based on the topic guide (Table S1), which was based on the adapted Health Belief Model and study objectives facilitating a systematic analysis of the key and recurring themes in the participants’ interviews. The framework evolved iteratively as new themes emerged during the data analysis, i.e., codes were revised, and new codes were added during the data analysis based on recurring patterns. The analysis was supported by NVivo software (version NVivo 12 (2018)) for categorising data under the identified themes and subthemes. A code sheet was developed from the first few source documents and developed into a master code sheet (Table S2). S.Z. and a research assistant independently coded the responses and thematically analysed the data to draw both descriptive and explanatory conclusions about the participants’ perceptions on the perceived benefits or barriers of the intervention and infant care practices. The study team familiarised themselves with the data by re-reading the transcripts to identify recurring issues, inconsistencies and possible categories, thus creating a coding framework and then coding using a mixture of deductive codes from the topic guide and inductive codes that emerged from the data, which were then applied to all the transcripts. The codes were then searched to identify themes, which were reviewed and defined. The coding process was an iterative process that involved changing and adding to them as more data became available. Participants were recruited to each of the three sample groups until it was considered that data saturation had been achieved, where no new information emerged from the additional IDIs. We expected to achieve data saturation after about 8 interviews [24].

All consent forms were kept in a secure locker. All recordings and transcriptions were kept on lockable devices that could only be accessed by the key research staff. All audio recordings were deleted as soon as the interviews were transcribed and no participant-identifiable information was written on the transcription sheets. No data were shared outside the research team.

## 3. Results

Thirty-three participants were interviewed across the three sample groups from different religions and geographical areas within Homa Bay County. This number achieved data saturation.

The age categories of participants are shown in Table 1. Amongst the 14 mothers/carers, 6 had not completed primary education, 5 had incomplete or had completed primary education, and 3 had completed secondary education; all were married except for 4, who were either single, separated or widowed. Three mothers/carers were housewives/unemployed, eight ran small-scale businesses and three were employed. Amongst the 12 Peer Mothers, 3 had primary education, 7 had incomplete high school, and 2 had completed high school or had higher-level education; all but 1 were currently married. The seven key informants included five study clinicians and two HCWs.

### 3.1. Process of Pro/Synbiotic Administration

During the initial few days following the recruitment into the trial, pro/synbiotic administration to newborns was performed almost solely by trained Peer Mothers and clinical officers. However, they would gradually pass on the responsibility to the mothers/carers by teaching them the safe and appropriate technique and eventually letting the mothers administer the pro/synbiotics under supervision. All three participant groups found the administration process of the pro/synbiotics relatively easy. Hygiene awareness during the administration among the Peer Mothers was very high, and the process was highly standardised, as described by one Peer Mother (Table 2, Quote 1).

#### 3.1.1. Administration of Pro/Synbiotics by Mothers

There were mixed views regarding whether the HCWs and Peer Mothers believed that the mothers/carers could administer the pro/synbiotics themselves. While most of the HCWs were positive that the process was easy enough for mothers/carers to administer the pro/synbiotics on their own, the Peer Mothers were more cautious. Nine (75%) Peer Mothers expressed concerns in terms of maintaining good hygiene standards and compliance in the absence of the trial staff. This conflict is demonstrated by the following two quotes: (Table 2: Quotes 2 and 3).

#### 3.1.2. Determining Factors for Method of Administration

The opinions on the preferred method of administration of the pro/synbiotics varied between all the participants. Most Peer Mothers and HCWs reported that their method of choice would vary on a case-by-case basis and were hesitant to settle on one specific method. However, most agreed that pouring the powder directly into the infants’ mouth was better tolerated with older infants, as described in the following two quotes: (Table 2: Quotes 4 and 5).

Both the HCWs and Peer Mothers found direct administration as the most convenient and reliable method for older infants (Table 2: Quote 6).

Six (42%) mothers/carers agreed that direct administration allowed for better confirmation that the complete dose was taken by their baby, as evidenced in the following two quotes: (Table 2: Quotes 7 and 8).

In contrast, some mothers/carers mentioned that their babies sometimes could struggle to consume the pro/synbiotic when given directly in powder form (Table 2: Quotes 9 and 10).

The importance of involving the mother/carer in decision-making regarding administration was highlighted by one HCW (Table 2: Quote 11).

However, four (33%) mothers stated that they were not able to express breastmilk, either due to stigma or low milk production (Table 2: Quote 12).

### 3.2. Perceived Benefits of Pro/Synbiotics and Participation in This Study

Four codes emerged that did not appear to be influenced by participant attributes, such as age, education and employment status.

#### 3.2.1. Accessible Healthcare and Education

This was the most coded node among the mothers/carers, with 13 (92.8%) mentioning that they were reassured that both themselves and the participating infants would receive medical care when needed, as it was stated as a benefit of study participation. Some of the mothers/carers stated that education on good infant care practices during the trial visits had a positive influence (Table 2: Quotes 13 and 14).

#### 3.2.2. Protection from Diseases

Twelve mothers/carers (86%) perceived that the pro/synbiotics protected the infants from diseases (Table 2: Quotes 15, 16, 17 and 18).

#### 3.2.3. Healthy Growth of the Infant

Nine mothers/carers (64%) stated that the pro/synbiotics helped their babies gain weight or made them stronger and more active (Table 2: Quotes 19, 20 and 21).

#### 3.2.4. Improved Appetite

This node was coded during seven (50%) of the mother/carer interviews (Table 2: Quotes 22 and 23).

### 3.3. Perceived Barriers to the Intervention

Commonly re-emerging codes were a lack of paternal involvement, work and other commitments, other people’s opinions, challenges in delivery and a lack of breastmilk. As with the perceived benefits, no correlations between the participant attributes (e.g., age, education or employment status, and number of children) and the codes were observed.

#### 3.3.1. Lack of Paternal Involvement

Two mothers (17%) mentioned that the fathers were absent during the consent process and did not have active involvement in taking care of the infants (Table 2: Quote 24).

In addition, two (29%) HCWs agreed that the lack of paternal involvement was a potential barrier to the uptake of the pro/synbiotics. The following quote explains the issues that may arise because of this and solutions to overcome this barrier: (Table 2: Quote 25).

#### 3.3.2. Work and Other Commitments

Five (42%) mothers found it difficult to stick to the dietary administration and study schedules due to work and other personal commitments (Table 2: Quote 26).

In addition, five (42%) Peer Mothers also acknowledged commitments as an issue that would affect the adherence to recommended breastfeeding practices. This was also a challenge when it came to carrying out their supportive roles, as it would sometimes affect the waiting times and inconvenience other household visits if the mother was not available at the scheduled visit (Table 2: Quote 27).

#### 3.3.3. Other Community Members’ Opinions

Four (33%) mothers and four (33%) Peer Mothers raised concerns over the negative opinions of the neighbours and other community members. They stated that they were told their children were being exploited by the research staff, who were conducting dangerous experiments on them (Table 2: Quotes 28 and 29).

However, reassurance by the trial staff and coping strategies of the mothers/carers towards these negative comments seemed to be good; some mothers said that they did not let these comments get to them and replied by saying that they were benefiting from the pro/synbiotics (Table 2: Quotes 30 and 31).

There were concerns from both the mothers and Peer Mothers who stated that neighbours who witnessed trial staff entering the participants’ homes with cooler boxes perceived that the pro/synbiotics were anti-retroviral (ARV) medication for managing the Human Immunodeficiency Virus (HIV) and spread rumours that the infant and the mother were HIV positive. However, none of the key informants raised this point as a potential barrier (Table 2: Quote 32).

#### 3.3.4. Challenges in the Method of Delivery

Key informants identified the HCW strikes and the COVID-19 pandemic as challenges to the data collection. These put strains on the health system in general, as well as the trial (Table 2: Quote 33 and 34).

However, these challenges were considered to mainly affect the study logistics rather than affect the community acceptability of the interventions and trial.

### 3.4. Infant Care Practices

The understanding of recommended breastfeeding practices was generally good among the mothers/carers. However, the understanding of the importance of breastfeeding practices seemed vague. Some mothers mentioned breastmilk protecting their babies from illnesses and helping them to grow stronger; however, most mothers said they practiced breastfeeding because breastmilk is the only type of food the baby can consume in the early stages of life, and they had a relatively poor understanding of the benefits of breastmilk (Table 2: Quote 35).

One carer mentioned the concept of connection with the baby while breastfeeding (Table 2: Quote 36).

The understanding of recommended WASH practices and the importance of hygiene were well understood by the mothers/carers (Table 2: Quotes 37 and 38).

Despite a good understanding of WASH practices, the Peer Mothers raised concerns over sometimes finding the infants in unsanitary conditions when they visited the households, especially when these visits were not expected (Table 2: Quote 39).

One Peer Mother mentioned that this might be because of inaccessibility to the required facilities and products, like soap, in order to maintain good standards of hygiene (Table 2: Quote 40).

## 4. Discussion

Overall, there was a high level of satisfaction and acceptability regarding the use of the pro/synbiotics among all the interviewed groups: mothers/carers, Peer Mothers and HCWs. The mothers/carers perceived healthy growth, the prevention of diseases and improved appetite as benefits of the pro/synbiotics. These findings are directly relevant to developing novel interventions to promote growth and development during early infancy and the period of exclusive breastfeeding, when the frequency of the onset of stunting is greatest [25].

This is consistent with previous findings of acceptability and perceived improved appetite and weight gain in the studies of dietary tools or nutrients in western Kenya. In a qualitative study in rural and urban communities in western Kenya, dietary tools, like slotted spoons, marked bowls, and illustrative counselling cards to improve maternal and infant nutrition, were well received by communities and mothers [26]. Similarly, a high acceptability in focus group discussions was reported amongst Luo families in western Kenya, adults and children, in a study of a micronutrient powder (Sprinkles) [27]. The acceptability of lipid-based nutrient supplements was also high in Haiti and Malawi, although this was provided for older infants and children [28,29].

However, it is important to note that mothers/carers cited that the support they received as a consequence of participating in the clinical trial was the greatest benefit, and the Peer Mothers concurred in their recognition of this factor. Although there was high acceptance amongst mothers/carers of the study procedures, some found it difficult to stick to the weekly visit schedule due to work and other commitments. This may impair scaling-up interventions that require frequent administration, especially amongst working mothers. Passing on the responsibility of pro/synbiotic administration to mothers/carers, after appropriate training and for those who feel confident, would reduce the number of scheduled visits and give mothers/carers more flexibility. However, compliance with pro/synbiotic administration would have to be monitored closely should pro/synbiotics be administered as a public health intervention.

This study also revealed varied views on the optimal method of pro/synbiotic administration. Most mothers/carers, Peer Mothers and HCWs agreed that it was easier to administer the pro/synbiotic directly into the mouth for older infants. For younger babies and some mothers, a lack of breastmilk or difficulties expressing breastmilk to mix with the pro/synbiotic was an issue. Overall, the HCWs reported that the mothers/carers were able to administer the pro/synbiotics themselves. However, the Peer Mothers raised concerns regarding the ability of mothers/carers to follow hygienic practice in the pro/synbiotic administration.

A main barrier to the pro/synbiotic administration was the negative influence of some community members. Extensive consultation with various stakeholders in Homa Bay County, including the Community Advisory Board, religious leaders, local administrative officers, Peer Mothers, Community Health Volunteers and Ministry of Health, through the county and sub-county health management teams was undertaken before this study started and also regularly throughout this study to answer any questions and respond to concerns. Our experience suggests that these concerted efforts to engage relevant stakeholders were critical to the success of the PROSYNK study. Similar stakeholder engagement would be required ahead of further research or use of pro/synbiotics as a public health intervention.

The HCWs also highlighted the challenges posed by a lack of paternal involvement. Although a lack of paternal involvement was a recurring code under potential barriers, in families where both parents were involved in the care of the infant, the mothers reported their partners’ involvement to be a positive and supportive factor. Engaging fathers in the consent for pro/synbiotic administration is likely important for both research and public health contexts.

Overall, the goal to reach saturation point was achieved under the various themes. On the other hand, despite the same codes re-emerging under the perceived barriers theme most of the time, focus group discussions or more key informant interviews may have identified new codes, as this theme had the most variety of answers given during the interviews compared with the other themes.

## 5. Limitations

We were careful when providing information for this qualitative research to explain that it was entirely separate from the main study. However, we cannot exclude the possibility that some mothers/carers may have felt intimidated or not fully at ease to share their honest feelings and opinions, as they may have perceived the interviewer to be part of the trial team, and any adverse comments may negatively affect their participation in the trial.

Additionally, having to translate the interview transcriptions from Swahili or Dholuo to English was a limitation. This meant that there was a possibility that some expressions that could not be directly translated might have been missed or misunderstood. This study was also conducted during the COVID-19 pandemic. Hence, the interviews with the HCWs were performed via WhatsApp calls. Following the interviews, the researcher noted that they found it harder to build rapport with the participants and that a lack of visual cues may have hindered the potential for gaining more insights.

There was also a concern over equivalence, as some key informant interviews were conducted by the researcher S.Z. in English, whereas all interviews with the mothers/carers and Peer Mothers were conducted in local languages by F.A. A difference in positionality between the two interviewers might have led to misinterpretations while comparing the code trends between different participant groups (e.g., perceived benefits expressed by the mothers vs. key informants).

Finally, in view of the limited qualitative research, as cited above, and likely variations in beliefs, attitudes and practices regarding infant nutrition, our findings may not be generalisable to other communities. Rather, qualitative research should be undertaken as an essential element of developing novel interventions in different contexts.

## 6. Conclusions

Pro/synbiotic administration may be an affordable, safe and effective public health intervention to protect gut health and, thereby, reduce childhood malnutrition and its associated adverse outcomes. This study showed very high satisfaction and acceptability among the community users (mothers/carers and Peer Mothers), as well as the HCWs regarding pro/synbiotic administration early in infancy and during exclusive breastfeeding. It also revealed key issues regarding stakeholder engagement and processes for the administration of pro/synbiotics to infants by the mothers/carers. Along with clinical and cost-effectiveness studies, further operational and qualitative research is required to explore ways to mitigate the barriers identified in this study.

## Figures and Tables

**Table 1:** Participant age groups by age category.

Participant Type	Age Group (Years)			Total N (%)
	20–34 N (%)	35–50 N (%)	>50N (%)	
Mothers/carers	10 (71.4)	2 (14.3)	2 (14.3)	14 (100)
Healthcare workers	2 (28.6)	3 (42.9)	2 (28.6)	7 (100)
Peer Mothers	7 (58.3)	4 (33.3)	1 (8.3)	12 (100)
Total	19	9	5	33

**Table 2:** Themes and participant quotes.

Theme	Quote	
Process of pro/synbiotic administration	Q1	“When you get to the house of a mother, you have to sanitize your hands, the cooler box carrying the supplement, after that, you clean it using a paper towel, after that you take another paper towel and cover the top of the cooler box because that is what you would use as the surface, then you put on your gloves, that is when you open the cooler, after opening it, you take the supplement and put it on the surface that you had set, then you wipe the cup that you are going to use, after that, you open the sup-plement and put it in the cup then if you are supposed to use sterile water you use, or the mother might express the breastmilk, but before that, she has to sanitize her hands and wash her hands on a running water, so that we can prevent germs from getting to the child”. (Peer Mother 006)
Administration of pro/synbiotics by mothers	Q2	“They can be administered by the mother themselves if the mother is enlightened and is taught and you have observe and you have done the demonstration in her presence; you do it, and then you allow her to do it in your presence, then you are sure that this mother will do it after she understands the importance of participating.” (MOH 001)
	Q3	“It is not easy because you don’t know what might happen when you leave for them the supplement, you cannot be sure whether the mother has given it or not. And maybe when you left it for them, they would want to taste how it is, at times her other child can unwrap it, so when you left it, we are not certain if they will give it because even with the medicines they are given you will find that there are some mothers that don’t give them to their children, that is why we go there and ensure that the child gets all of it and take everything back to the facility”. (Peer Mother 002)
Determining factors for method of administration	Q4	“Personally, I don’t have a preferred method that I will use. But I will just say at an early stage between the first 10 days, it’s always hard to administer the supplement directly to the mouth. In that event, it actually chokes the participants. that is where we prefer using sterile water and expressed breast milk during the first 10 days of life on up to three weeks of life. So, it’s okay at that point.” (HCW 005)
	Q5	“So, in terms of delivering them, I use the baby’s age. Because when they are still young, maybe at enrolment on day one or day two, delivering it as powder at times becomes tricky. So, it’s easier when you mix it with sterile water, but then at around the age of between four and five to six weeks, that’s when we can now start giving it di-rectly to the baby’s mouth. So personally, I use both but in terms of the age of the baby.” (HCW 002)
	Q6	“The method that we have at the moment is the best, administering it in the mouth directly, because that will convince us for sure that the infant has taken.” (MOH 001)
	Q7	“I am choosing [direct administration] because, when placed in the bottle, you may find that there are some remains in the bottle”. (Mother 001, IDI)
	Q8	“… when it is not mixed, there are some content of the medicine that remains on the bottle”. (Mother 010)
	Q9	“With the one given directly, at times she finds it hard to consume it.” (Mother 007)
	Q10	“When given directly, at times [the supplement] can stick on the throat and it is hard to digest.” (Mother 011)
	Q11	“The mother sometimes would suggest, and whenever they suggest, we go with what they suggested. Sometimes you go to administer the supplement and the mother would ask you to just give them the cup, so they can express breast milk and give it to the infant. You see, in that case, then the mother’s preference takes precedence, we just give it to the mother, the mother expresses and then gives it to the child. Yeah, so mother’s preference also is a determining factor apart from age.” (HCW 001)
	Q12	“There was another way we were told to use but I still dint have breast milk, we were told to use breast milk but since I did not have enough milk we were told to just use water.” (Mother 003)
Perceived benefits of pro/synbiotics and participation in this study	Q13	“All I know is that since we got to the study, it has really helped me and made things easy for me because the child might be sick, and you are there to help out, so I have found everything so easy that even my husband feels the same way, so that is what I can say and what I have seen”. (Mother 004)
	Q14	“Since she got into this study, it has really helped me, I can say it has really helped me a lot, because there was a time she could have fever, cold and there were also things that I dint know like how to take care of the baby’s umbilical cord, how to breastfeed, how to handle the baby and my own cleanliness, I dint know but you taught me … They also taught me how to bond with the baby well, how to dispose the baby’s diapers, and if in case you see danger signs when you should rush the baby to the hospital.” (Mother 001)
	Q15	“It prevents things like measles and worms that might be in her stomach”. (Mother 003)
	Q16	“In my mind I believe that the supplement prevents some diseases that might affect the child”. (Mother 004)
	Q17	“I have not seen my child contracting these normal diseases that children tend to have and her body is also good so those are the benefits that I can say”. (Mother 007)
	Q18	“The difference that I am seeing between him and my other children is that the other ones were getting sick especially the age that this one is, but with him ever since I got to this study I have not seen him getting sick, he is healthy.” (Mother 008)
	Q19	“It makes the child strong, it keeps the baby healthy, the baby grows well”. (Carer 002)
	Q20	“It helps the baby grow in the right way”. (Mother 006)
	Q21	“when you compare it with other children, you find that they are less active and weak compared to this one who is active and strong”. (Mother 013)
	Q22	“I can say it looks like something that provides the baby with vitamin and gives the child appetite making the child to breast feed well, so it doesn’t give you hard time, that is what I am seeing with the supplement”. (Mother 004)
	Q23	“they started giving my child medicine and my child is breastfeeding really well”. (Mother 008)
Perceived barriers to the intervention	Q24	“[The father] is not saying anything because he doesn’t know that I am in this study so he just always sees people coming in the house”. (Mother 012)
	Q25	“The major challenge comes during introduction of the supplements to these par-ticipants is that; majority of the mothers who come to the facility don’t accompany themselves with their husbands. So, what you find is only a mother who has delivered in the ward. You will find the mother and the baby to consent but we tend to forget about the father. So it would be my advice that even if the father might be away, there is a role, a key role they play, so it would be prudent enough so they would be involved in this process, Because you find yourself, you introduce the study or the process to the mother, the mother agrees, but when they go home and they take supplements at one point, the father feels like he’s not part of the team. So that is one challenge—at any point we are introducing such an investigation of products, the two parties should be well informed. And if it’s a minor, the mother or the carer should be well informed about the procedures or activities that are ongoing.” (HCW 004)
	Q26	“I just think it’s just work, that is the thing that would make me find it difficult in following the rules of the study, I don’t see any other, because that is the only thing that would make me be away from the child”. (Mother 004)
	Q27	“I have not seen any major challenge, is just that at times when we go for the visits you will find out that some people are not there, so you have to wait and at the same time you have to go and visit some other people that is where I find it challenging”. (Peer Mother 011)
	Q28	“The thing I have found difficult is the opinion of others, relatives, friends, they say what will they benefit from you and what would you think of a person who takes full responsibility of your child, so they have not taken it as a good thing, yes they talk.” (Mother 013)
	Q29	“[A friend’s husband] was saying that the KEMRI [Kenya Medical Research Institute] only do the research with the child’s stomach and my baby will die.” (Mother 003)
	Q30	“If they come with the negative comments, I explain to them what I know about the study and how I was taught before I got to the study”. (Mother 013)
	Q31	“With me, I am just okay because you cannot stop someone saying whatever they want to say, I just concluded that even if they want to talk let them talk, there is no problem”. (Mother 014)
	Q32	“Initially, [the neighbours] thought that this food that we were giving were ARVs, so [the mother] would prefer you to hide it somewhere so that you can give the child because their neighbours would ask why the child is given medicine on a daily basis. So that is what we have tried to eradicate by telling them that it is food and not medicine, they were afraid at the beginning but now I see that they are okay”. (Peer Mother 008)
	Q33	“We’ve had several strikes, where the Ministry of Health staff was on strike. And that is a challenge to admission, and also the process that is required in the PROSYNK study. We also had stockouts, at times tests are required, but we are not able to do. We’ve been transferring the PROSYNK study’s patients, especially those who don’t require admission, to private hospitals and follow them up there for better care. And even in tests that are not able to be done within the hospital because of supply stock-outs, we’ve always gone to private laboratories, and requested the test to be done. And that is a big challenge that we’ve experienced in the PROSYNK study.” (MOH 002)
	Q34	“So, the challenges that we’ve experienced so far is mainly with regard to con-ducting clinical trials in COVID-19 times. So that is top. Because right from when we started, there are instances where, like, at the moment, there is a surge, … we’ve di-vided the group into cohorts. A lot of planning and management has gone into this just to ensure that we always have a team in case there is exposure. This has also affected our recruitment, because when we are not working at full capacity, then we are not able to get participants. It could also complicate building follow ups. So, I think COVID-19 has really been something else for conduct of clinical trials.” (HCW 001)
Infant care practices	Q35	“The reason why the child is being breastfed is because that is the child’s food, just like you also, ugali is your food that even if you don’t take it you don’t feel comfortable, so I breastfeed the baby because that is his food that is on his mind.” (Mother 004)
	Q36	“Breast milk is stronger than that of the cow because that is just a cow but the mother has connection with the baby.” (Carer 005)
	Q37	“Hygiene is important so that she cannot contract diseases which may make her have things like stomach ache”. (Mother 006)
	Q38	“First for me, I have to ensure that his clothes are clean, his washed clothes are dry, secondly I as a mother who is breastfeeding, when I go somewhere and come back, If I have not bathe then I should clean my breast before breastfeeding, the environment should also be clean even if in any case someone wants to carry the baby, he/she should wash their hands or sanitize so that he/she doesn’t handle the baby with dirt”. (Mother 004)
	Q39	“The places that we are visiting, it is not true that everybody is clean, some are clean and others are not. At times you visit and the mother has come out from the toilet and you find she will not remember to wash her hands, instead she will just go ahead and give [the supplement to the baby].” (Peer Mother 003)
	Q40	“At times you find that when you go to some places you may find that it is difficult for the participant to even get soap, so to practice hygiene and cleanliness of her sur-roundings is hard so that maybe one of the causes”. (Peer Mother 006)

## Data Availability

All data produced in the present study are available upon reasonable request to the authors.

## Supplementary Materials

The following supporting information can be downloaded at: https://www.mdpi.com/article/10.3390/nu17030495/s1, Table S1a: Topic Guide for semi-structured interviews with mothers and carers; Table S1b: Topic Guide for semi-structured interviews with healthcare workers (HCWs) and Peer Mothers; Table S2: Coding Framework.

## Abbreviations

The following abbreviations are used in this manuscript:

EED	Environmental enteric dysfunction
HCW	Healthcare worker
IDI	In-depth interview
PROSYNK	PRObiotics and SYNbiotics in infants in Kenya
WASH	Water, sanitation and hygiene
