# Supplementary material for: Probiotics and Synbiotics Administered to Young Infants: Perceptions and Acceptability Amongst Carers and Healthcare Workers in Western Kenya
Source: Nutrients. 2025 Jan 29;17(3):495. doi: 10.3390/nu17030495 (PMC11820114; doi:10.3390/nu17030495)
Supplement: Supplementary file 1 [file nutrients-17-00495-s001.zip › nutrients-3428811-supplementary.pdf]

Probiotics and synbiotics administered to young infants: perceptions and acceptability amongst carers and healthcare workers in Western Kenya.

Otiti MI et al.

Supplementary materials

Table S1a: Topic Guide for semi-structured interviews with mothers and carers

**Topics**		**Prompts**
Introduction and background	Introductions	• Introduce yourself<br>• Learn who they are in relation to the infant: mother, Peer Mother etc.<br>• Build rapport<br>• Verbal consent
	Information about the infant	• How old is the infant?<br>• Are they healthy? Do they have any medical conditions? Are they on any medication?
	Trial participation	• When were they enrolled in the study?
	Social context	• Who the baby lives with etc.
Experiences of using pro/synbiotic supplements for infants	Adherence	• Did they find it easy to adhere to the trial schedule?<br>• Have they missed any doses and if yes why?<br>• What do they think have been the greatest barriers to compliance?
	Storage	• Do they think the capsules are ergonomic and easy to store? (Do not spend too much time on this prompt.)
	Mode of delivery	• Do they find administration easy?<br>• What is their preferred method of administration?<br>• Do they think they could easily administer the supplements themselves?<br>• Which method do they prefer (mixing it with formula/breast milk in a clean container or sprinkling directly into the infant's mouth) and why?
	Satisfaction	• Have they noticed any benefits?<br>• Have they noticed any negative effects? E.g., vomiting<br>• What have they found most helpful during their participation in the trial in regard to the supplements?<br>• What have they found most difficult<br>• during their participation in the trial in regard to the supplements?
Infant care practices	Breastfeeding and	• Are they breastfeeding or using formula milk?

among mothers/carers in the community that protect against infections	sanitation	• How important is breastfeeding to them?
	Hygiene & sanitation	• What hygiene and sanitation measures are they taking to protect the infant?<br>• How important are hygiene and sanitation for them in regards to the infant's health?
	Effectiveness of dietary supplements	• How important/useful/beneficial do they think the dietary supplements are in protecting their infant's health?
Other/additional		• Do they have any suggestions for improvement?

Table S1b: Topic Guide for semi-structured interviews with healthcare workers (HCWs) and Peer Mothers

**Topics**		**Prompts**
Introduction and background	Introductions	• Introduce yourself<br>• Build rapport<br>• Verbal consent
	Role in participants' care	• Their professional title and qualifications<br>• How are they involved in the infant or mother's care?<br>• How often do they see the mother and/or infant?
Perceptions and experiences of using pro/synbiotic supplements for infants	Adherence	• What do they think are the greatest barriers to adherence/compliance?<br>• What have they tried/suggest in order to overcome these barriers?
	Storage	• Do they think the capsules are ergonomic and easy to store? (Do not spend too much time on<br>• this prompt.)
	Mode of delivery	• Do they find administration easy?<br>• What is their preferred method of administration (mixing it with formula/breast milk in a clean container or sprinkling directly into the infant's mouth) and why?<br>• What are the determinants in choosing the mode of delivery?<br>• Do they think supplements can be easily administered by the<br>• mothers/carers?
	Satisfaction	• Have they noticed any benefits?<br>• Have they noticed any negative effects? E.g., vomiting<br>• How do they think the general satisfaction among mothers and carers is?
Infant care practices	Breastfeeding and sanitation	• How important do they think breastfeeding is in regard to infant's health?<br>• What do they advise their patients in terms of breastfeeding?
	Hygiene & sanitation	• How important do they think hygiene and sanitation are in regard to infant's health?<br>• What do they advise their patients in terms of hygiene and sanitation?
	Effectiveness of dietary supplements	• How important/useful/beneficial do they think the dietary supplements are in protecting their infant's health?
Other/additional		• Do they have any suggestions for improvement?

Table S2: Coding Framework

Introduction
HCWs and Peer mothers
Role and experience
Mothers
enrolment date
infant's age
infant's health
living situation
Can mothers give supplement themselves?
Determining factors for mode of administration
Infant care practices
Breastfeeding
Breastfeeding practices
Importance of breastfeeding
Recommendations on breastfeeding
Dietary supplements
Importance of dietary supplements
WASH
Importance of WASH
Recommendations on WASH
WASH practices
Perceived barriers - challenges
Beliefs
Cleanliness
Compliance
Consent
COVID
Delivery
Education
Fear
Fear of expressing milk
Health system
Lack of breastmilk
Lack of paternal involvement
